# Modelling vibro–acoustic response of lightweight square aluminium panel influenced by sound source locations for active control

**DOI:** 10.1038/s41598-022-14951-y

**Published:** 2022-06-24

**Authors:** Chukwuemeke William Isaac, Stanislaw Wrona, Marek Pawelczyk, Hamid Reza Karimi

**Affiliations:** 1 Department of Machine Technology, Faculty of Mechanical Engineering, Silesian University of Technology, Konarskiego 18A, 44-100 Gliwice, Poland; 2 Department of Measurements and Control Systems, Silesian University of Technology, Akademicka 16, 44-100 Gliwice, Poland; 3 Department of Mechanical Engineering, Politecnico di Milano, 20156 Milan, Italy

**Keywords:** Mechanical engineering, Aerospace engineering

## Abstract

This paper uses numerical approach to give insight into the structural–acoustic response of a lightweight square aluminium panel. It takes into consideration different locations of a primary sound source in an acoustic medium and how these locations influence the response of the structural panel. Finite element method as well as the first-order deformation theory are employed for constructing the numerical model. Experimental measurements of the mode shapes and velocity frequency response of the vibrating panel are used to validate the results of the finite element model. Furthermore, vibro-acoustic emission indexes such as sound transmission loss, sound pressure level and far-field directivity of sound pressure are obtained numerically. The results show that different locations of the primary sound source significantly influence the response of the structural panel to reduce noise. Sound source typically positioned close to the structural panel lowers the efficiency of the vibrating panel to reduce noise. Moreover, the sound distribution profiles at the radiated end of the vibrating panel for the different locations of the sound source are investigated. The study shows that the variation of the zones of quiet, vibro-acoustic emission parameters and sound distribution profiles obtained can provide vital information about the best positioning of structural source for both active vibration and noise control.

## Introduction

Lightweight structural panels such as shells and thin plates are increasingly becoming attractive for noise reduction/control particularly in the automotive, aerospace, marine and building industries. Recently, researchers have found these structural panels particularly convenient for use in active noise and vibration control. Apart from their relatively good vibro–acoustic response in low frequency noise reduction, their reduced weights make them handy and can easily be incorporated into the design of windows and household appliances such as the washing machines, refrigerators as well as many food processing devices. In practice, these structural panels encounter some unexpected working conditions due to the location where the primary source of noise emanates. Consequently, the structural and acoustic responses of these panels to effectively reduce noise are compromised. It is, therefore, of utmost importance to investigate different locations of generated noise and how these noise source locations influence the response of the lightweight panel to control or/and reduce noise. A good understanding of this investigation will provide researchers vital information of the correct placement of secondary sound sources utilized for noise cancellation during active vibration and noise control.

Secondary sources for active structural and acoustic control such as structural actuators or electrodynamic shakers and also sensors, microphones or other noise-cancellation devices have been placed on or around vibrating panels to cancel noise generated from primary sound sources. In recent years, different optimization methods have been carried out to find more effective ways to locate actuators and sensors on the vibrating structural^1,2^. Also, control sources^3^ and microphones^4^ in the acoustic field in line with the primary source, have been used to provide information about noise control results. However, a number of factors have contributed to the challenge of properly placing these secondary sources on or around the structural panel. The separation distance between secondary sources, number of secondary sources, distance of secondary sources to vibrating panel, to mention a few, are some factors contributing to the effectiveness of noise cancellation. These factors become more pronounced when dealing with low frequency noise which are experienced during outdoor vehicular movements and from noise generated industrial machines.

For vibro-acoustic problems at low frequency regions, structural panels with reduced weight (i.e., thin-walled structures) have the tendencies to produce low transmission loss due to mass law effect^5^. To address this challenge, various structural lightweight panels made from composites^6^, smart material^7^, functionally graded material^8^ as well as active metallic material^9^, have been improved and successfully used for active noise and vibration control. Authors have also demonstrated that the vibro-acoustic emission indices which include the sound transmission loss, radiated sound efficiency, sound power, far-field sound pressure, etc., are active indicators that reliably show how efficient structural panels respond to sound waves^10^. These panels admit incident sound waves, transmit these waves through their thicknesses and then radiate them at their radiating surface. Several parameters have also been identified to affect these emission indexes. For example, geometric parameters such as shell thickness^11^, finite or infinite dimensions of panels^12^ as well as boundary conditions (BC) of the vibrating structure^13^, have been widely investigated to understand their influences on their vibro-acoustic emission. Other parameters which are associated with the material properties (i.e., stiffness, Mach number, etc.)^14,15^ and structural properties^16^ have also been investigated.

Several analytical solutions have been developed to handle vibro-acoustic problems in noise reducing casing^17^ and for problems involving structural–acoustic bounded and unbounded domains. However, numerical approximations, for example, the finite element method (FEM)^18^ has continued to be a more effective tool for modelling structural–acoustic problems. This method could be combined with other methods, for example, boundary element method^19^, to give more robust solution especially for the coupled structural–acoustic interface. However, this unified method is still computationally burdensome. To this end, efforts are currently being put together to address the limitations of computational efficiency. Cui et al.^20^, combined both the edged-based smoothed FEM as well as the gradient-weighted FEM to obtain more reliable solution of structural–acoustic problems. Also, Song and Wolf^21^, developed a scale boundary FEM which was implemented by Lehman et al.^22^ and recently by Li et al.^23^ to solve both coupled bounded and unbounded vibro-acoustic problems.

On the investigation and analysis of vibro-acoustic problems performed by most of the authors mentioned above, a noise source located at a fixed point in the structural–acoustic region was used to produce the sound waves. Attention, however, was seldom given to the locations where the generated primary sound waves emanate. In the present study, the authors seek to find answers to how different locations of a noise generating loudspeaker, positioned inside a finite rigid casing, influences the vibro-acoustic response of a vibrating lightweight square aluminium panel. One of the challenges of active vibration and noise control is to obtain the best locations of the secondary sound source with respect to the primary sound wave distribution and spectrum. A good understanding of the different locations of the emanating sound waves, as described in this study, will help in effective positioning of actuators, sensors, microphones or other active noise cancellation device, on or around the vibrating panel. The study first uses finite element method with the shear deformation theory to solve the structural and acoustic responses of the bounded domain problems. Numerical results of various vibro-acoustic emission indexes such as the sound transmission loss $(STL)$, sound pressure distribution as well as the far-field directivity of the sound pressure are obtained, and the effect of the varying sound source locations on these indexes are analysed in the mid-low frequency range (i.e., 0–1000 Hz). Lastly, the study examines the influence of source locations on the far-field directivity pattern of the vibrating panel with fully clamped and simply supported boundary conditions.

## Model and problem description

**Figure 1:**
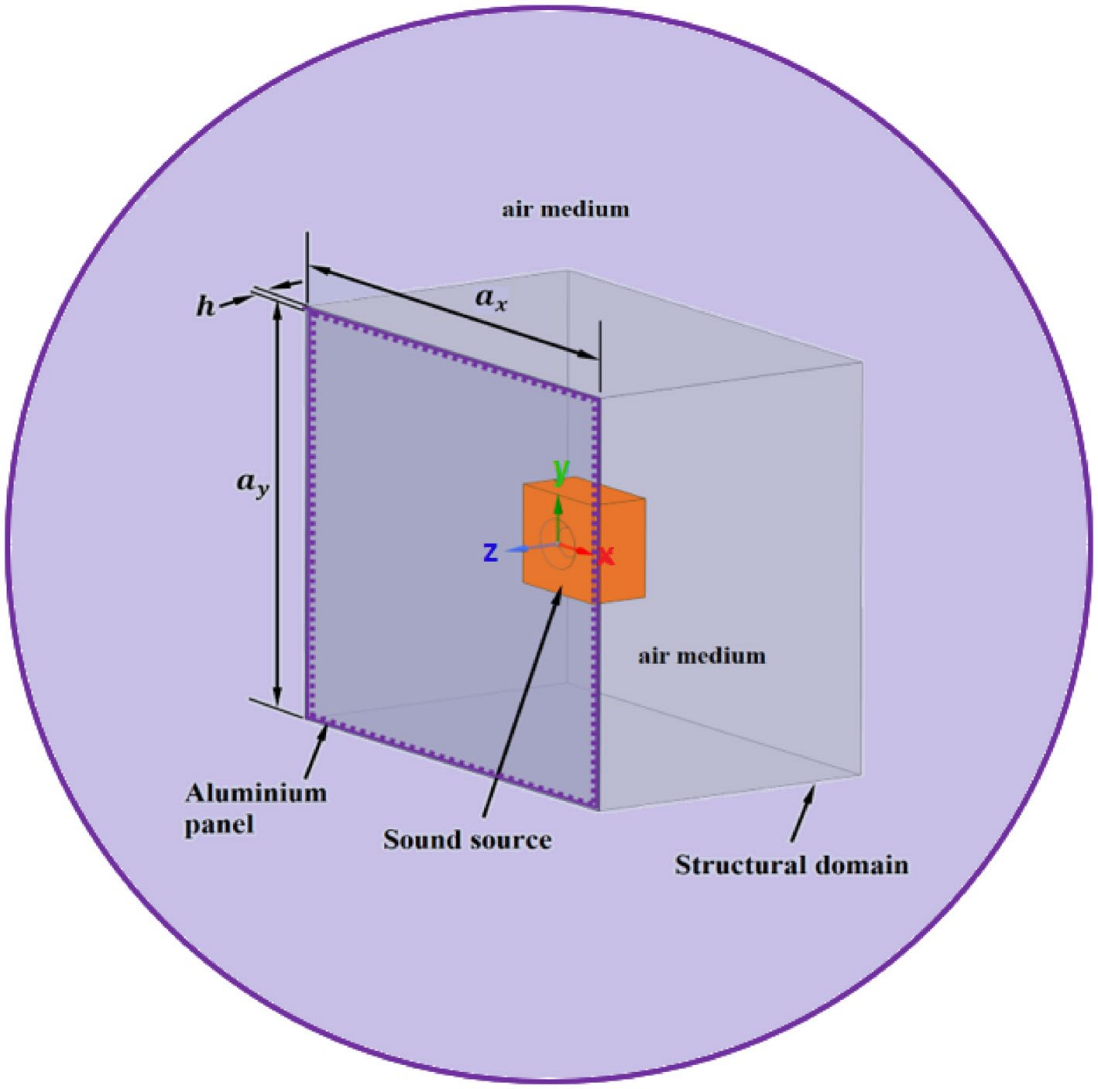
Schematic representation of the vibro-acoustic problem for the present study.

**Figure 2:**
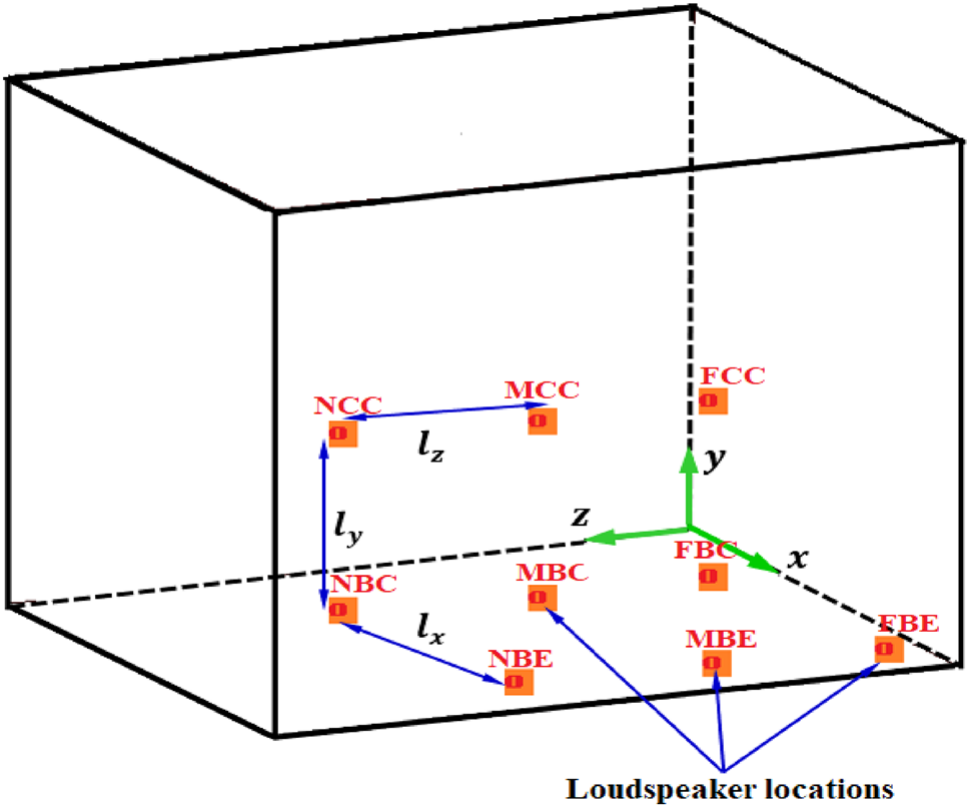
Representation of various designated locations of loudspeaker inside a rigid casing.

The model of this study includes an isotropic aluminium thin-walled panel such that its thickness ($h$) is far less than both its length (${a}_{x}$) and its width (${a}_{y}$)^24^, with dimensions ${a}_{x}=420 \; \text{mm}$, ${a}_{y}=420 \; \text{mm}$ and $h=1 \; \text{mm}$, as shown in Fig. 1. The rationale for choosing these dimensions is to advance the research performed by the authors^4^ especially for further investigation of active noise and vibration control. A fully clamped structural panel is placed inside a space enclosure of infinite dimension bounded by an acoustic domain. The bounded domain in the near field region consists of the structural domain defined by the cartesian coordinate $(x,y,z)$ and an air domain while the enclosed room of infinite dimension is defined by a spherical coordinate in the far-field region. Sound source emanating from a loudspeaker is used to excite the aluminium panel which in turn radiates sound waves. In this study, the loudspeaker is placed at nine different locations inside a rigid casing which also houses the lightweight square aluminium panel. For the sake of convenience, these nine different locations of the sound source are designated thus: near centre (NCC), mid centre (MCC), far centre (FCC), near bottom centre (NBC), mid bottom centre (MBC), far bottom centre (FBC), near bottom edge (NBE), mid bottom edge (MBE) and far bottom edge (FBE). Figure 2 shows a schematic representation of the different designated locations of the loudspeaker producing the sound wave. The distances along the $x$, $y$ and $z$ axes from one designated location point to another location point are denoted as ${l}_{x}$, ${l}_{y}$ and ${l}_{z}$, respectively. It is assumed that the rigid casing is perfectly absorbing, hence, the vibro-acoustic results will be the same for any volume dimension of rigid casings.

## Structural modelling

### Theoretical formulation

For the aluminium square panel with dimensions ${a}_{x}$, ${a}_{y}$ and $h$ as represented in Fig. 1, at the reference surface, the first-order shear deformation theory is used to obtain the displacements as follows

1a $$\mathcal{U}\left(x,y,z, t\right)=\mathcalligra{u}\left(x,y,t\right)-z\left({\alpha }_{x}\right)$$

1b $$\mathcal{V}\left(x,y,z,t\right)=\mathcalligra{v}\left(x,y,t\right)-z\left({\alpha }_{y}\right)$$

1c $$\mathcal{W}\left(x,y,z,t\right)=\mathcalligra{w}\left(x,y,t\right)$$

where the symbols $\mathcal{U}$, $\mathcal{V}$ and $\mathcal{W}$ are the displacements along $x$, $y$ and $z$ directions at time $\left(t\right)$ variable, respectively. Also, $\mathcalligra{u}$, $\mathcalligra{v}$ and $\mathcalligra{w}$ are the panel mid-plane displacements in the $x$, $y$ and $z$ directions, respectively. Furthermore, notations ${\alpha }_{x}=\partial \mathcalligra{w}/\partial x(x,y,t)$ and ${\alpha }_{y}=\partial \mathcalligra{w}/\partial y(x,y,t)$, are the rotations of the transverse normal about $y$ and $x$ axes, respectively. It is assumed that there are no twist changes for the vibrating square panel, hence, the normal strains (i.e., ${\varepsilon }_{x}$ and ${\varepsilon }_{y}$) and shear strain (i.e., ${\gamma }_{xy}$) along the mid-plane can be expressed as

2a $${\varepsilon }_{x}={\varepsilon }_{x}^{0}-z{\psi }_{x} , { \psi }_{x}=\frac{{\partial }^{2}\mathcalligra{w}}{\partial {x}^{2}}$$

2b $${\varepsilon }_{y}={\varepsilon }_{y}^{0}-z{\psi }_{y} , {\psi }_{y}=\frac{{\partial }^{2}\mathcalligra{w}}{\partial {y}^{2}}$$

2c $${\gamma }_{xy}={\gamma }_{xy}^{0}-z{\psi }_{xy} , {\psi }_{xy}=2\frac{{\partial }^{2}\mathcalligra{w}}{\partial x\partial y}$$

where ${\varepsilon }_{x}^{0}=\partial \mathcalligra{u}/\partial x$ and ${\varepsilon }_{y}^{0}=\partial \mathcalligra{v}/\partial y$ represent the normal strains while ${\gamma }_{xy}^{0}=\partial \mathcalligra{u}/\partial y+\partial \mathcalligra{v}/\partial x$ is the shear strain in the reference surface. Also, the terms ${\psi }_{x}$, ${\psi }_{y}$ and ${\psi }_{xy}$ are the middle surface curvature changes. The transverse shearing strain components ${\gamma }_{xz}$ and ${\gamma }_{yz}$ are assumed to be zero due to the thin-walled nature of the aluminium panel. The resultant stress $\sigma (x,y)$ comprises of both the force ($Q$) and moment ($M$) resultants which can be expressed in components form as

3 $$\left\{\begin{array}{c}{Q}_{x}\\ {Q}_{y}\\ {Q}_{xy}\end{array}\right\}={A}^{k}\left[\begin{array}{ccc}1& \mu & 0\\ \mu & 0& 0\\ 0& 0& \frac{1-\mu }{2}\end{array}\right]\left\{\begin{array}{c}{\varepsilon }_{x}^{0}\\ {\varepsilon }_{y}^{0}\\ {\gamma }_{xy}^{0}\end{array}\right\}+{D}^{k}\left[\begin{array}{ccc}1& \mu & 0\\ \mu & 0& 0\\ 0& 0& \frac{1-\mu }{2}\end{array}\right]\left\{\begin{array}{c}-{\psi }_{x}\\ -{\psi }_{y}\\ -{\psi }_{xy}\end{array}\right\}$$

4 $$\left\{\begin{array}{c}{M}_{x}\\ {M}_{y}\\ {M}_{xy}\end{array}\right\}={D}^{k}\left[\begin{array}{ccc}1& \mu & 0\\ \mu & 0& 0\\ 0& 0& \frac{1-\mu }{2}\end{array}\right]\left\{\begin{array}{c}{\varepsilon }_{x}^{0}\\ {\varepsilon }_{y}^{0}\\ {\gamma }_{xy}^{0}\end{array}\right\}+{F}^{k}\left[\begin{array}{ccc}1& \mu & 0\\ \mu & 0& 0\\ 0& 0& \frac{1-\mu }{2}\end{array}\right]\left\{\begin{array}{c}-{\psi }_{x}\\ -{\psi }_{y}\\ -{\psi }_{xy}\end{array}\right\}$$

where ${A}^{k}$, ${D}^{k}$ and ${F}^{k}$ are the stiffness coefficients of the thin-walled panel, respectively expressed as

5a $${A}_{ij}^{k}={\int }_{-\frac{h}{2}}^\frac{h}{2} \frac{E(z)}{ 1-{\mu }^{2}(z)}dz$$

5b $${D}_{ij}^{k}={\int }_{-\frac{h}{2}}^\frac{h}{2} \frac{\mu (z)E(z)}{ 1-{\mu }^{2}(z)}dz$$

5c $${F}_{ij}^{k}={\int }_{-\frac{h}{2}}^\frac{h}{2} \frac{{\mu }^{2}(z)E(z)}{ 1-{\mu }^{2}(z)}dz$$

The values of the material parameters $E$ and $\mu$ are given in Table 1. Moreover, the total energy functional (${\mathbf{E}}_{T}$), can be expressed in terms of the energy due to strain (${{\varvec{S}}}_{\varepsilon }$) and due to motion (${{\varvec{T}}}_{E}$), expressed as^25^

**Table 1:** Properties and dimensions of the aluminium material utilized for the present study.

Property/dimension	Symbol (unit)	Value
Young’s modulus	$E \; (\text{GPa})$	70
Poisson ratio	$\mu$	0.3
Density	$\rho \; (\text{kg}/\text{m}^{2})$	2770
Length	${a}_{x} \; (\text{mm})$	420
Width	${a}_{y} \; (\text{mm})$	420
Thickness	$h \;(\text{mm})$	1

6 $${\mathbf{E}}_{T}={{\varvec{S}}}_{\varepsilon }-{{\varvec{T}}}_{E}$$

where ${{\varvec{S}}}_{\varepsilon }$ over the area of the square panel can be expressed as

7 $${{\varvec{S}}}_{\varepsilon }= \frac{1}{2}{\int }_{0}^{{a}_{x}}{\int }_{0}^{{a}_{y}}{(Q}_{x}{\varepsilon }_{x}+ {Q}_{y}{\varepsilon }_{y}+ {Q}_{xy}{\gamma }_{xy})dxdy$$

Also, the kinetic energy ${{\varvec{T}}}_{E}$, of the square panel is written as

8 $${{\varvec{T}}}_{E}= \frac{1}{2}{\int }_{0}^{{a}_{x}}{\int }_{0}^{{a}_{y}}\left[{I}_{0}\left({\dot{\mathcalligra{u}}}^{2}+{\dot{\mathcalligra{v}}}^{2}+{\dot{\mathcalligra{w}}}^{2}\right)+{I}_{2}\left({\dot{{\alpha }_{x}}}^{2}+{\dot{{\alpha }_{y}}}^{2}\right)+2{I}_{1}(\dot{\mathcalligra{u}}\dot{{\alpha }_{x}}+\dot{\mathcalligra{v}}\dot{{\alpha }_{y}})\right]dxdy$$

where $\dot{\mathcalligra{u}}$, $\dot{\mathcalligra{v}}$ and $\dot{\mathcalligra{w}}$ represent the velocities at the mid-surface while the symbols ${I}_{0}$, ${I}_{1}$ and ${I}_{2}$ represent the moment of inertial defined respectively as

9a $${I}_{0}={\int }_{-\frac{h}{2}}^\frac{h}{2} \rho \left(z\right)dz $$

9b $${I}_{1}={\int }_{-\frac{h}{2}}^\frac{h}{2}z \rho \left(z\right)dz $$

9c $${I}_{2}={\int }_{-\frac{h}{2}}^\frac{h}{2} {z}^{2}\rho \left(z\right)dz $$

By applying the principle of virtual displacement, the governing equation of motion for the square panel can be obtained which is expressed as

10a $$-{\omega }^{2}\left[{{\varvec{M}}}_{{\varvec{p}}}\right]\left\{\mathcalligra{u}\right\}+\left[{{\varvec{K}}}_{{\varvec{p}}}\right]\left\{\mathcalligra{u}\right\}=0$$

where the matrices $\left[{{\varvec{M}}}_{{\varvec{p}}}\right]$ and $\left[{{\varvec{K}}}_{{\varvec{p}}}\right]$ represent the mass and stiffness of the vibrating panel, respectively. Also, the vector $\left\{\mathcalligra{u}\right\}$ is the displacement field and the symbol $\omega$ denotes the natural frequency. With the time function substituted into Eq. (10a), the equation of motion becomes

10b $$\left(\left[{{\varvec{K}}}_{{\varvec{p}}}\right]-{\omega }^{2}\left[{{\varvec{M}}}_{{\varvec{p}}}\right]\right)\left\{\mathcalligra{u}\right\}=0$$

Applying the Rayleigh damping equation ${C}_{p}={a}_{M}\left[{M}_{p}\right]+{b}_{K}\left[{K}_{p}\right]$ to Eq. (10b), where ${a}_{M}$ and ${b}_{k}$ are the damping coefficients for the mass and stiffness matrices, respectively. The numerical values of ${a}_{M}$ and ${b}_{K}$ used in this work are 3.8500347 and 0.0013203, respectively calculated from the first natural frequency. The governing equation of motion of the vibrating panel in the time domain becomes

10c $$\left[{{\varvec{M}}}_{{\varvec{p}}}\right]\left\{\ddot{\mathcalligra{u}}\right\}+\left[{{\varvec{C}}}_{{\varvec{p}}}\right]\left\{\dot{\mathcalligra{u}}\right\}+\left[{{\varvec{K}}}_{{\varvec{p}}}\right]\left\{\mathcalligra{u}\right\}=0$$

### Finite element formulations of the structural–acoustic bounded domain

#### Discretized equations of lightweight structural panel

At the midplane of the square thin-walled panel, a set of nodes ${x}_{k}\left\{k=1,\dots ,\zeta \right\}$ can be used to obtain the finite element parts. The expression of the displacement takes the following form

11 $$\mathcalligra{u}=\left[\begin{array}{c}{\mathcalligra{u}} \\ {\mathcalligra{v}} \\ {\mathcalligra{w}} \\ {\psi }_{x}\\ {\psi }_{y}\end{array}\right]=\sum_{k=1}^{\zeta }{\mathbb{N}}_{k}\left[\begin{array}{c}{\mathcalligra{u}} \\ {\mathcalligra{v}} \\ {\mathcalligra{w}} \\ {\psi }_{x}\\ {\psi }_{y}\end{array}\right]{e}^{i\omega t}=\sum_{k=1}^{\zeta }{\mathbb{N}}_{k}\left(x\right){\mathcalligra{u}}^{{\varvec{T}}}{e}^{i\omega t}$$

where ${\mathbb{N}}_{k}$ represent the standard displacement shape functions. Since the shearing component of the transverse displacement of the square panel is neglected, the bending component ($\mathcalligra{w}$) over its domain is given as

12 $$\mathcalligra{w}(x,y)={\mathbb{N}}_{k}^{T}\Delta \mathcalligra{w}$$

where $\Delta \mathcalligra{w}$ is the bending degree of freedom vector. The stiffness matrix ${\varvec{K}}$ has two components ${{\varvec{K}}}^{e}$ and ${{\varvec{K}}}^{b}$ which are the stiffnesses due to extension and bending, respectively. It is given as

13 $${\varvec{K}}={{\varvec{K}}}^{e}+{{\varvec{K}}}^{b}$$

Over the domain, these stiffness matrices are respectively discretized as

14a $${{\varvec{K}}}_{ks}^{e}={\int }_{\Omega }{{\varvec{B}}}_{k}^{{e}^{T}}{\varvec{D}}{{\varvec{B}}}_{s}^{e}d\Omega $$

14b $${{\varvec{K}}}_{ks}^{b}={\int }_{\Omega }{{\varvec{B}}}_{k}^{{b}^{T}}{\varvec{A}}{{\varvec{B}}}_{s}^{b}d\Omega +{\int }_{\Omega }{{\varvec{B}}}_{k}^{{b}^{T}}{\varvec{B}}{{\varvec{B}}}_{s}^{e}d\Omega +{\int }_{\Omega }{{\varvec{B}}}_{k}^{{e}^{T}}{\varvec{B}}{{\varvec{B}}}_{s}^{b}d\Omega $$

where the notation ${{\varvec{B}}}^{e}$ represents the extensional strain matrix and the notation ${{\varvec{B}}}^{b}$ represents the strain matrix due to bending. These terms are defined as

15 $${{\varvec{B}}}_{k}^{e}=\left[\begin{array}{ccccc}0& 0& 0& \frac{\partial {\mathbb{N}}_{k}}{\partial x}& 0\\ 0& 0& 0& 0& \frac{\partial {\mathbb{N}}_{k}}{\partial y}\\ 0& 0& 0& \frac{\partial {\mathbb{N}}_{k}}{\partial y}& \frac{\partial {\mathbb{N}}_{k}}{\partial x}\end{array}\right]$$

16 $${{\varvec{B}}}_{k}^{b}=\left[\begin{array}{ccccc}\frac{\partial {\mathbb{N}}_{k}}{\partial x}& 0& 0& 0& 0\\ 0& \frac{\partial {\mathbb{N}}_{k}}{\partial y}& 0& 0& 0\\ \frac{\partial {\mathbb{N}}_{k}}{\partial y}& \frac{\partial {\mathbb{N}}_{k}}{\partial x}& 0& 0& 0\end{array}\right]$$

Moreover, the expression of the mass matrix over the domain ($\Omega$) of the structural panel is

17 $${{\varvec{M}}}_{ks}={\int }_{\Omega }{{\varvec{G}}}_{k}^{T}\mathcalligra{m}{\boldsymbol{ }{\varvec{G}}}_{s}d\Omega $$

where

18 $${{\varvec{G}}}_{{\varvec{k}}}=\left[\begin{array}{ccccc}{\mathbb{N}}_{k}& 0& 0& 0& 0\\ 0& {\mathbb{N}}_{k}& 0& 0& 0\\ 0& 0& {\mathbb{N}}_{k}& 0& 0\\ 0& 0& 0& {\mathbb{N}}_{k}& 0\\ 0& 0& 0& 0& {\mathbb{N}}_{k}\end{array}\right]$$

and

19 $$\mathcalligra{m}=\left[\begin{array}{ccccc}{I}_{0}& 0& 0& {I}_{1}& 0\\ 0& {I}_{0}& 0& 0& {I}_{1}\\ 0& 0& {I}_{0}& 0& 0\\ {I}_{1}& 0& 0& {I}_{2}& 0\\ 0& {I}_{1}& 0& 0& {I}_{2}\end{array}\right]$$

With the help of numerical procedures, the matrix terms ${\varvec{A}}$, ${\varvec{B}}$, ${\varvec{D}}$ and $\mathcalligra{m}$ can be conveniently solved and the terms ${{\varvec{K}}}^{e}$, ${{\varvec{K}}}^{b}$ and ${\varvec{M}}$ can be approximated using Gauss integration method.

#### Discretized equations of the enclosed air domain

The acoustic fluid in the rigid cubic casing is air. Discretization of the enclosed air region is achieved by using a regular hexahedral element. The nodal pressure ${p}^{e}$ which acts on each node of the element can be written as

20 $${p}^{e}\left(x,y,z\right)={\mathbb{N}}_{f}\left(x,y,z\right){{\varvec{P}}}^{e}$$

where ${{\varvec{P}}}^{e}$ is the nodal pressure vector. The pressure gradient is written as

21 $${\nabla p}^{e}\left(x,y,z\right)={{\left[\frac{\partial }{\partial x} \frac{\partial }{\partial y} \frac{\partial }{\partial z}\right]}^{T}}{\mathbb{N}}_{f}\left(x,y,z\right){{\varvec{P}}}^{e}$$

with nodal shape functions, ${\mathbb{N}}_{f}=\left[{\mathbb{N}}_{1} {\mathbb{N}}_{2}\dots {\mathbb{N}}_{8}\right]$. Given the boundary of the elemental air domain (${\Omega }_{f})$, the elementary mass matrix in the enclosed air region is defined as

22 $${{\varvec{M}}}_{f}^{e}=\frac{1}{{{\rho }_{0}c}_{0}^{2}}{\int }_{{\Omega }_{f}}{{\mathbb{N}}_{f}}^{T}{\mathbb{N}}_{f}{J}_{f}d{\Omega }_{f}$$

where the notation ${\rho }_{0}$ represent the density of air, ${c}_{0}$ is the speed of sound in air and ${J}_{f}$ is the determinant of the Jacobian for air. Also, in the enclosed air region, the elementary stiffness matrix can be defined as

23 $${{\varvec{K}}}_{f}^{e}=\frac{1}{{\rho }_{0}}{\int }_{{\Omega }_{f}}{{{\varvec{B}}}_{f}}^{T}{{\varvec{B}}}_{f}{J}_{f}d{\Omega }_{f}$$

with

24 $${{\varvec{B}}}_{f}={{\left[\frac{\partial }{\partial x} \frac{\partial }{\partial y} \frac{\partial }{\partial z}\right]}^{T}}{\mathbb{N}}_f$$

#### Discretized equations of fluid–structure coupled domain

The coupled region of the enclosed structural-air domain is the interface between the enclosure region of the rigid casing and the internal wall of the rigid casing. The interface element with domain, ${\Omega }_{sf}$, is bounded by four nodes having two degrees of freedom on each of the nodes. The discretized elemental displacement in relation to the cubic shape function can be expressed as

25 $${\mathcalligra{u}}^{e}={\mathbb{N}}_{ui}{{\varvec{u}}}_{i}^{e}, \quad \quad  i=1, 2, 3, 4$$

where ${\mathbb{N}}_{ui}$ is the cubic shape function of the elemental normal displacement. The interfacial acoustic pressure acting on the four nodes of the element is written as

26 $${p}^{e}={\mathbb{N}}_{fi}{{p}_{i}^{e}}^{T}, \quad i=1, 2, 3, 4$$

where the notation ${\mathbb{N}}_{fi}$ signifies the linear shape functions obtained at each node of the element. The elementary coupling matrix (${\mathcal{K}}_{cf}^{e}$) bounded by ${\Omega }_{sf}$ is associated with the cubic and linear shape functions which can now take the form

27 $${\mathcal{K}}_{cf}^{e}={\int }_{{\Omega }_{sf}}{{\mathbb{N}}_{ui}}^{T}{\mathbb{N}}_{fi}{J}_{f}d{\Omega }_{sf}$$

## Acoustic modelling

### Theoretical formulation of the acoustic fields

As stated already, the rigid casing houses both the sound source and the lightweight aluminium panel which are all mounted inside an enclosed room. The sound wave from the loudspeaker incidents on the panel surface, reflects the wave, transmits sound wave through the thickness and then radiates the sound wave from the radiating surface of the panel. Within this linear acoustic domain, the equation governing the propagation of wave in terms of the velocity potential ($\phi$), is expressed as

28 $${\nabla }^{2}\phi -\frac{1}{{c}_{0}^{2}}\frac{{\partial }^{2}\phi }{\partial {t}^{2}}=0$$

where $\phi$ is the main variable used for the acoustic formulation. Also, the gradient operator $\nabla$ in Eq. (28) is given in Eq. (21). The dynamic pressure is given as

29 $$P=-{\rho }_{0}\frac{\partial \phi }{\partial t}$$

The relationship between the velocity potential and the velocity of the acoustic fluid particle is ${\varvec{\upnu}}=\nabla \phi$ while the velocity Neumann BC is also related in the form ${n}_{0}^{T}\nabla \phi ={{\varvec{\upnu}}}_{n}$, where ${n}_{0}$ and ${{\varvec{\upnu}}}_{n}$ are the outward unit normal vector of the acoustic domain and the outward normal velocity, respectively. By combining the time harmonic excitation of Eq. (28) with time dependence (${e}^{j\omega t}$), the Helmholtz wave equation becomes

30 $${\nabla }^{2}\phi -\frac{{\omega }^{2}}{{c}_{0}^{2}}\phi =0$$

In the linear acoustic domain, three acoustic pressure waves exist which are expressed as

31a $${P}^{i}(x,y,z)={A}_{0}^{i}{e}^{j\omega t-{\eta }_{ix}x-{\eta }_{iy}y-{\eta }_{iz}z}$$

31b $${P}^{r}(x,y,z)={A}_{0}^{r}{e}^{j\omega t-{\eta }_{ix}x-{\eta }_{iy}y-{\eta }_{iz}z}$$

31c $${P}^{t}(x,y,z)={A}_{0}^{t}{e}^{j\omega t-{\eta }_{ix}x-{\eta }_{iy}y-{\eta }_{iz}z}$$

where ${P}^{i}$ is the incident pressure wave, ${P}^{r}$ is the reflected pressure wave and ${P}^{t}$ indicates the transmitted pressure wave. Note that the corresponding symbols ${A}_{0}^{i}$, ${A}_{0}^{r}$ and ${A}_{0}^{t}$ of these pressure waves as described in Eq. (31) are the amplitudes of the respective pressure waves. Also, ${\eta }_{ix}$, ${\eta }_{iy}$ and ${\eta }_{iz}$ indicate the respective wavenumbers in the direction of $x$, $y$ and $z$ axes.

### Discretized equations of the acoustic far-field surface domain

**Figure 3:**
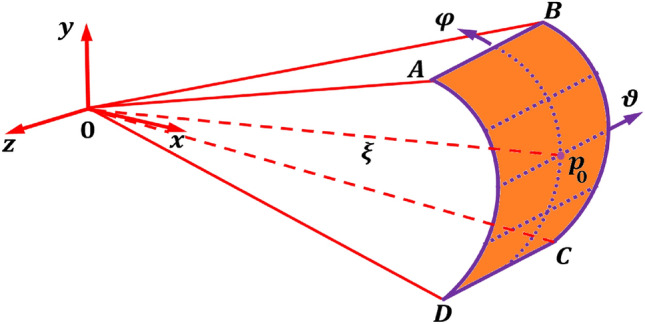
Discretized model of the acoustic far-field spherical bounded domain with a far-field point ${p}_{0}$.

In this section, finite element is used to discretize the boundary of the acoustic far-field. Consider a far-field point $${p}_{0}$$ on the discretized surface ABCD with $$n$$ number of nodes as shown in Fig. 3. A non- dimensional axis $$(\xi )$$, i.e., line segment $$O{p}_{0}$$, is assumed to connect the cartesian coordinate ($$x,y,z)$$ at the origin $$O$$ to point $${p}_{0}$$ on the surface having a far-field coordinate ($$\xi ,\vartheta ,\varphi )$$, an arbitrary coordinate ($$\widehat{x},\widehat{y},\widehat{z})$$ and a nodal coordinate ($${\varvec{x}},{\varvec{y}},{\varvec{z}})$$. For the $$n$$ nodes discretized surface ABCD, the nodal coordinates at point $${p}_{0}$$ can be interpolated by means of the shape functions $${\mathbb{N}}_{a}\left(\vartheta ,\varphi \right)$$ in the far-field coordinates described according to the relations

32a $$x\left(\xi ,\vartheta ,\varphi \right)= \xi .{\mathbb{N}}_{a}\left(\vartheta ,\varphi \right){\varvec{x}}$$

32b $$y\left(\xi ,\vartheta ,\varphi \right)= \xi .{\mathbb{N}}_{a}\left(\vartheta ,\varphi \right){\varvec{y}}$$

32c $$z\left(\xi ,\vartheta ,\varphi \right)= \xi .{\mathbb{N}}_{a}\left(\vartheta ,\varphi \right){\varvec{z}}$$

where ${\mathbb{N}}_{a}=\left[{\mathbb{N}}_{1}, {\mathbb{N}}_{2},\dots , {\mathbb{N}}_{n}\right]$, are the shape functions in the far-field coordinate $\vartheta$ and $\varphi$ of the discretized surface ABCD. In the far-field subdomain of Fig. 3, the acoustic pressure amplitude can be discretized as

33 $$P\left(\xi ,\vartheta ,\varphi ,t\right)= {\mathbb{N}}_{a}\left(\vartheta ,\varphi \right){P}_{a}\left(\xi \right){e}^{j\omega t}$$

where ${P}_{a}$ is the pressure amplitude in the direction of the non- dimensional axis. In the same vein, the nodal velocity potential in the far-field subdomain of the surface finite element is obtained by means of the shape functions ${\mathbb{N}}_{a}$ in the far-field co-ordinate $\vartheta$ and $\varphi$, given as

34 $$\phi \left(x,y,z\right)= {\mathbb{N}}_{a}\left(\vartheta ,\varphi \right){\phi }_{a}\left(\xi \right)$$

where ${\phi }_{a}$ is the velocity potential at all points in the direction $\xi$. At the far-field point ${p}_{0}$, the gradient operator $\nabla$ of the Helmholtz equation i.e., Eq. (28), can be rewritten as^26^)

35 $$\nabla = {{\varvec{b}}}_{a}^{1}\frac{\partial }{\partial \xi }+\frac{1}{\xi }\left({{\varvec{b}}}_{a}^{2}\frac{\partial }{\partial \vartheta }+{{\varvec{b}}}_{a}^{3}\frac{\partial }{\partial \varphi }\right)$$

where the vectors ${{\varvec{b}}}_{a}^{1}$, ${{\varvec{b}}}_{a}^{2}$ and ${{\varvec{b}}}_{a}^{3}$ are derived from the coordinate ($\widehat{x},\widehat{y},\widehat{z})$ of the arbitrary point ${p}_{0}$ on surface ABCD and are given in components form as

36a $${{\varvec{b}}}_{a}^{1}=\frac{1}{{J}_{a}}\left[\begin{array}{c}{\widehat{y}}_{22}{\widehat{z}}_{33}-{\widehat{z}}_{23}{\widehat{y}}_{32}\\ {\widehat{z}}_{23}{\widehat{x}}_{31}-{\widehat{x}}_{21}{\widehat{z}}_{33}\\ {\widehat{x}}_{21}{\widehat{y}}_{32}-{\widehat{y}}_{22}{\widehat{x}}_{31}\end{array}\right]$$

36b $${{\varvec{b}}}_{a}^{2}=\frac{1}{{J}_{a}}\left[\begin{array}{c}{\widehat{z}}_{13}{\widehat{y}}_{32}-{\widehat{y}}_{12}{\widehat{z}}_{33}\\ {\widehat{x}}_{11}{\widehat{z}}_{33}-{\widehat{z}}_{13}{\widehat{x}}_{31}\\ {\widehat{y}}_{12}{\widehat{x}}_{31}-{\widehat{x}}_{11}{\widehat{y}}_{32}\end{array}\right]$$

36c $${{\varvec{b}}}_{a}^{3}=\frac{1}{{J}_{a}}\left[\begin{array}{c}{\widehat{y}}_{12}{\widehat{z}}_{23}-{\widehat{z}}_{13}{\widehat{y}}_{22}\\ {\widehat{z}}_{13}{\widehat{x}}_{21}-{\widehat{x}}_{11}{\widehat{z}}_{23}\\ {\widehat{x}}_{11}{\widehat{y}}_{22}-{\widehat{y}}_{12}{\widehat{x}}_{21}\end{array}\right]$$

where the term ${J}_{a}$ is obtained by evaluating the determinant of the Jacobian at ${p}_{0}$. The coefficient matrices for the surface domain (${\Omega }_{s})$, in the far-field co-ordinate $\vartheta$ and $\varphi$ with constant $\xi$ are given as

37a $${\mathcal{F}}_{a}^{0}={\int }_{{\Omega }_{s}}{{\varvec{B}}}_{a}^{{1}^{T}}{{\varvec{B}}}_{a}^{1}{J}_{a}d{\Omega }_{s}$$

37b $${\mathcal{F}}_{a}^{2}={\int }_{{\Omega }_{s}}{{\varvec{B}}}_{a}^{{2}^{T}}{{\varvec{B}}}_{a}^{2}{J}_{a}d{\Omega }_{s}$$

37c $${\mathcal{F}}_{a}^{1}={\int }_{{\Omega }_{s}}{{\varvec{B}}}_{a}^{{2}^{T}}{{\varvec{B}}}_{a}^{1}{J}_{a}d{\Omega }_{s}$$

37d $${\mathcal{M}}_{a}^{0}=\frac{1}{{c}_{0}^{2}}{\int }_{{\Omega }_{s}}{\mathbb{N}}_{a}^{T}{\mathbb{N}}_{a}{J}_{a}d{\Omega }_{s}$$

with their shape function relationships expressed as ${{\varvec{B}}}_{a}^{1}={{\varvec{b}}}_{a}^{1}{\mathbb{N}}_{a}$ and ${{\varvec{B}}}_{a}^{2}={{\varvec{b}}}_{a}^{2}{\mathbb{N}}_{a,\vartheta }+{{\varvec{b}}}_{a}^{3}{\mathbb{N}}_{a,\varphi }$.

### Vibro-acoustic emission parameters

In this section, one of the objectives is to obtain some emission parameters such as the sound pressure of the acoustic medium, radiated sound power and sound transmission loss. The effects of loudspeaker positions on some of these emission indices are discussed in the numerical results section.

#### Far-field sound pressure

**Figure 4:**
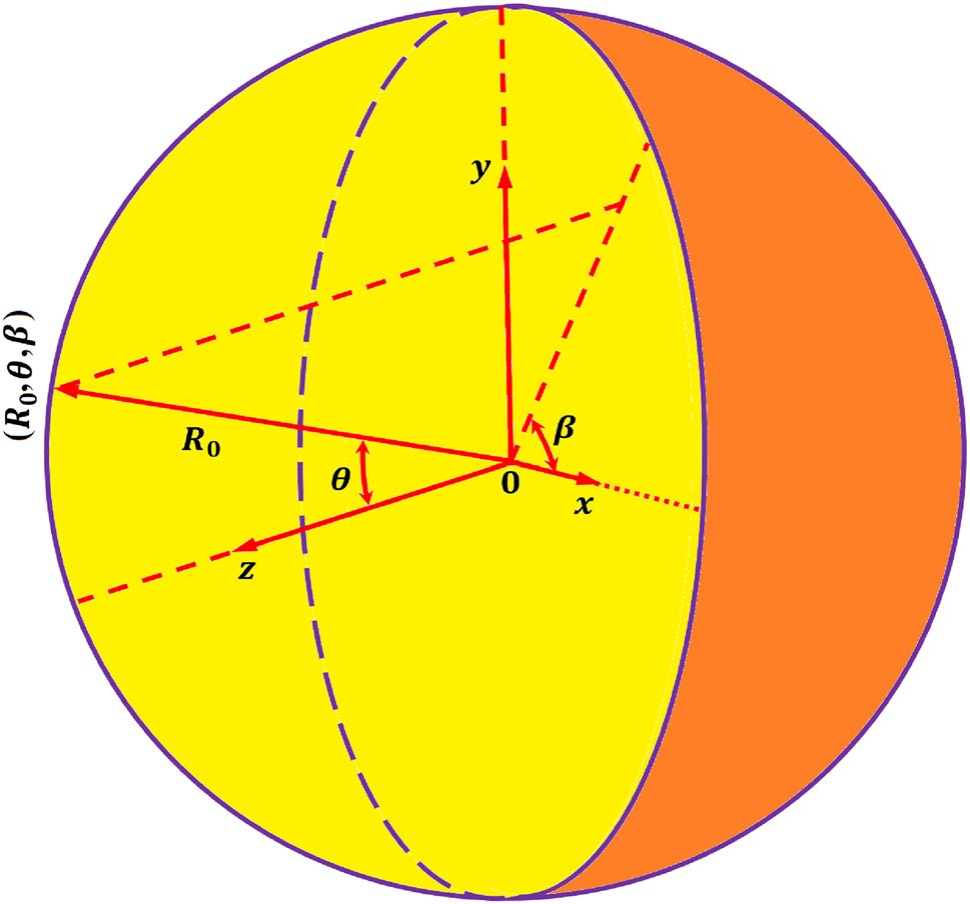
Representation of the coordinate system for the spherical enclosed acoustic domain.

Figure 4 shows a spherical enclosed acoustic domain with origin $$o$$ of the Cartesian coordinate system $$(x$$,$$y,z)$$ transformed to the spherical coordinate system $$({R}_{0}$$,$$\theta ,\beta )$$ along a far-field distance $${R}_{0}$$. The far-field sound pressure amplitude at this receiving point $$({R}_{0}$$,$$\theta ,\beta )$$ in spherical coordinate can be expressed as^27^

38 $${P}_{f}({R}_{0},\theta ,\beta ) =j{\rho }_{0}\omega \frac{{e}^{-j\eta {R}_{0}}{{\varvec{\upnu}}}_{n}}{2\pi {R}_{0}}$$

where ${{\varvec{\upnu}}}_{n}$ is the normal velocity due to the position vector of the elemental surface of the panel position at point $o$ and the acoustic wavenumber symbol $\eta =\omega /{c}_{0}$. Let ${\lambda }_{a}=\eta cos\theta sin\beta$ and ${\lambda }_{b}=\eta sin\theta sin\beta$ be the respective trace wavenumbers along the $x$ and $y$ axes; then the velocity response of the vibrating structural panel is given as

39 $${{\varvec{\upnu}}}_{{\varvec{p}}}({\lambda }_{a},{\lambda }_{b};\omega ) ={\int }_{S}{{\varvec{\upnu}}}_{n}(x,y){e}^{j({\lambda }_{a}x+{\lambda }_{b}y)}dS$$

#### Radiated sound power

Given a defined point ($${r}_{0})$$ located on the face sheet of the structural panel to a receiving point ($${r}_{f})$$, the sound power transmitted as a result of the excitation of the panel is expressed as^28^

40 $${\Psi }_{t}=\frac{1}{2}Re{\int }_{S}{{\varvec{\upnu}}}_{{\varvec{p}}}^{\boldsymbol{*}}\left({r}_{0}\right){P}^{t}({r}_{f})dS$$

The symbols $Re$ and superscript * of ${\varvec{\upnu}}$, denote the real part of sound intensity and the complex conjugate, respectively. It is worthwhile to note that the velocity in Eq. (40) obtained from Eq. (39) can also be derived from the nodal displacement of the finite element solution as described in the preceding section. Let the reference power be ${\Psi }_{ref}$, then the sound pressure level can be easily expressed as

41 $$SPL=20\mathrm{log}\frac{{\Psi }_{t}}{{\Psi }_{ref}}$$

The relationship between the radiated sound power along a far-field distance ${R}_{0}$ and the far-field sound pressure are approximated as^29^

42 $${\Psi }_{{R}_{0}}= {\int }_{0}^{\pi /2}{\int }_{0}^{2\pi }\frac{{\left|{P}_{f}({R}_{0},\theta ,\beta )\right|}^{2}}{2{\rho }_{0}{c}_{0}}{R}_{0}sin\beta d\theta d\beta $$

#### Sound transmission loss

A very important acoustic emission parameter that is often utilised to determine the capacity of a vibrating panel to reduce noise is the sound transmission loss. It is calculated in terms of the ratio of the incident to transmitted sound powers. In order to evaluate this parameter, the transmission coefficient $(\tau )$ of the structural panel is first obtained by dividing the transmitted power $({\Psi }_{t})$ by the incident power $\left({\Psi }_{i}\right)$ given as

43 $$\tau =\frac{{\Psi }_{t}}{{\Psi }_{i}}$$

where the incident power of the lightweight square aluminium panel can be expressed as

44 $${\Psi }_{i}=\frac{{P}^{{i}^{2}}{a}_{x}{a}_{y}\mathrm{cos}\theta }{2{\rho }_{0}{c}_{0}}$$

with the transmission loss coefficient given in Eq. (43), the sound transmission loss of the vibrating structural panel is conveniently expressed as

45 $$STL=10{\mathrm{log}}_{10}\left(\frac{1}{\tau }\right)$$

## Validation of model

**Figure 5:**
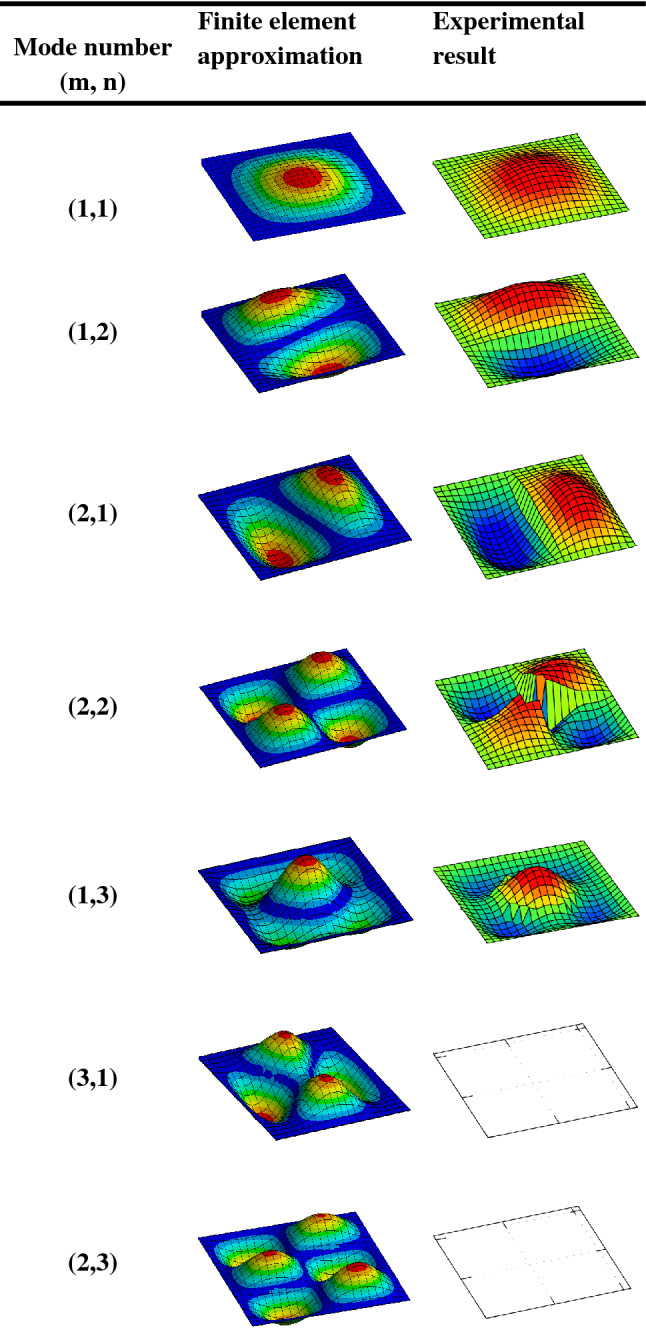

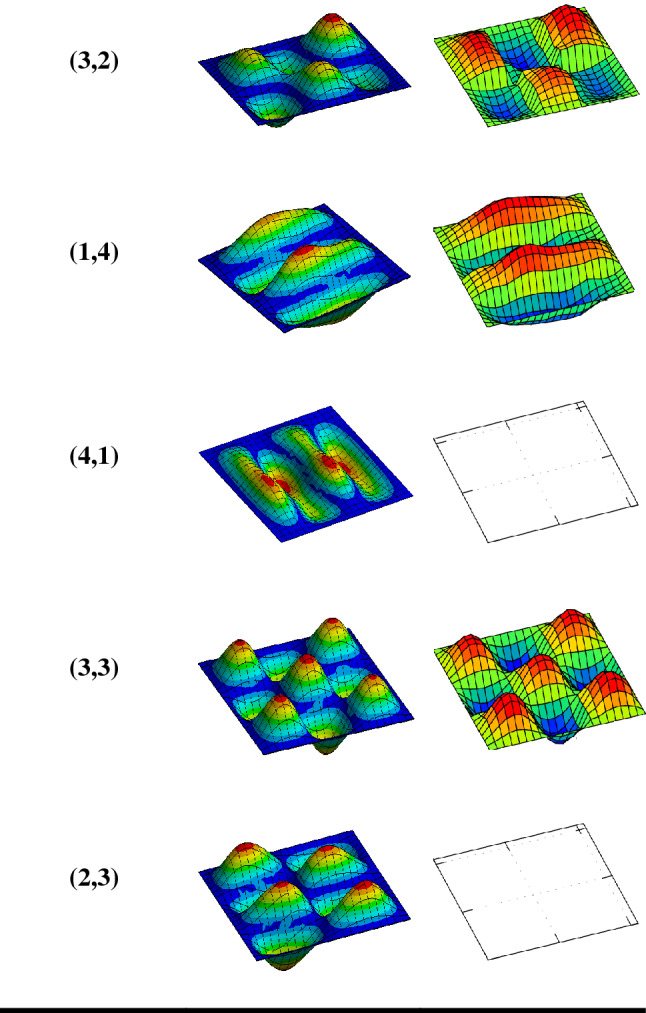
First twelve mode shapes comparison between finite element approximation and measured results for the aluminium panel excited by a loudspeaker placed at FBC source location.

**Figure 6:**
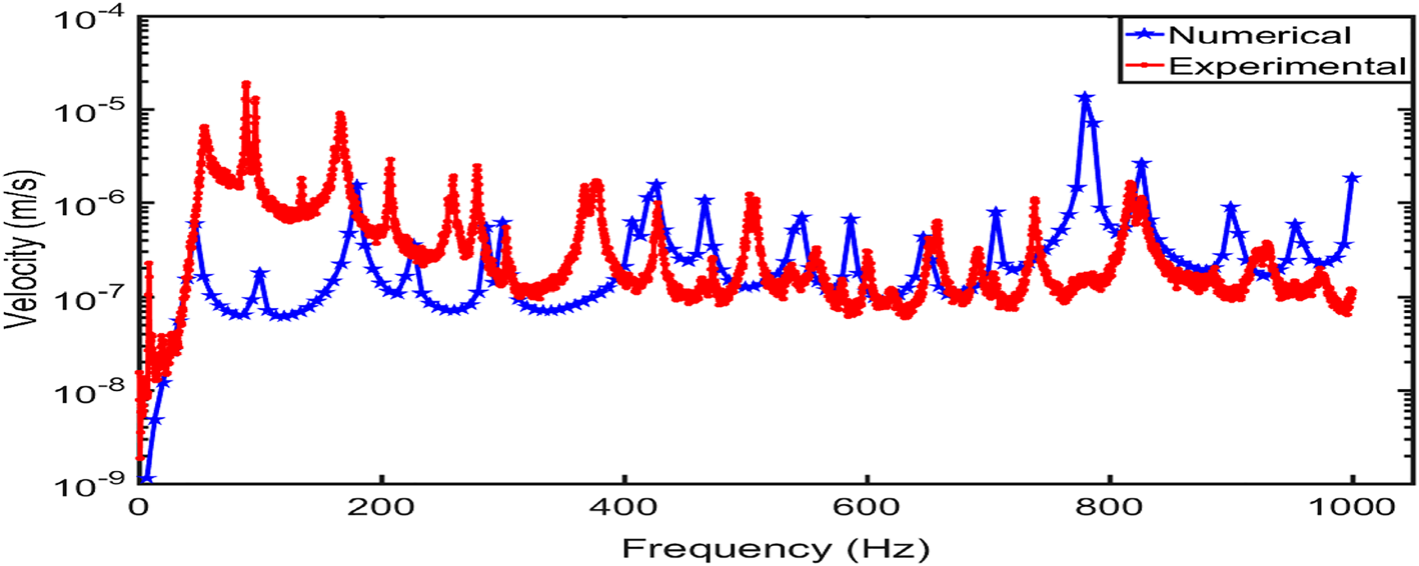
Velocity-frequency response comparison between finite element approximation and experimental measurement with CCCC boundary conditions.

Experimental measurements were taken and utilized for the validation of the finite element solutions. Both the measured and approximation solutions adopted the same parameters and fully clamped boundary conditions. The experiment has already been performed by the same authors with full description of the set-up^8^. Also, the implementation of the finite element approximation was performed using the ANSYS simulation tool. Figure 5 depicts the first twelve mode shapes of the square aluminium panel acoustically excited by the acoustic sounds emanating from a loudspeaker placed inside a rigid casing. In the experimental results, it is observed that mode numbers six (3,1), seven (2,3), ten (4,1) and twelve (2,4) are not distinguishable because they are weakly excited by the loudspeaker at source location FBC. In the vibro-acoustic problem, the radiated sound wave by the square aluminium panel is directly proportional to the velocity at which the square panel vibrates. Figure 6 compares the velocity frequency responses between the numerical approximation and experimental measurement. These curves are plotted on the log scale which shows similar trends with each other. However, the initial slightly higher amplitudes from the experimental results could be attributed to the uneven excitation of the loudspeaker. The numerical results show more response behaviour of the square panel than the experimental results. For example, the frequency response curve of the numerical approximation shows the possibility of the aluminium panel to be excited at the same natural frequency, but different magnitudes as depicted in Table 2.

**Table 2:** Comparison between the natural frequency obtained numerically and experimentally with CCCC boundary condition.

Mode number	Natural frequency (Hz)	
	Numerical	Experimental
(1,1)	44.7	54.0
(1,2)	97.6	88.0
(2,1)	97.6	95.5
(2,2)	146.1	134.0
(1,3)	175.9	166.0
(3,1)	178.3	–
(2,3)	224.4	–
(3,2)	224.4	207.5
(1,4)	286.2	259.0
(4,1)	286.3	–
(3,3)	300.7	278.0
(2,3)	331.6	–

To further verify the numerical result of the fully clamped (CCCC) square aluminium panel, a simply supported (SSSS) square aluminium panel is modelled with the same parametric conditions. The fully clamped model has the conditions on its boundary as follows

46a $$\mathcalligra{u}= \mathcalligra{v}=\mathcalligra{w}={\alpha }_{x}={\alpha }_{y}=0 \;\; \mathrm{at} \;\;x = 0, \;\;{a}_{x}$$

46b $$\mathcalligra{u}= \mathcalligra{v}=\mathcalligra{w}={\alpha }_{x}={\alpha }_{y}=0 \;\;\mathrm{at} \;\; y = 0, \;\;{a}_{y}$$

For the modelled square aluminium panel with SSSS, the conditions on its boundary are

47a $$\mathcalligra{u}= \mathcalligra{v}=\mathcalligra{w}={\alpha }_{y}=0; {M}_{x}=0 \;\; \mathrm{at} \;\;x = 0, \;\; {a}_{x}$$

47b $$\mathcalligra{u}= \mathcalligra{v}=\mathcalligra{w}={\alpha }_{x}=0; {M}_{y}=0 \;\; \mathrm{at} \;\; y = 0, \;\; {a}_{y}$$

**Figure 7:**
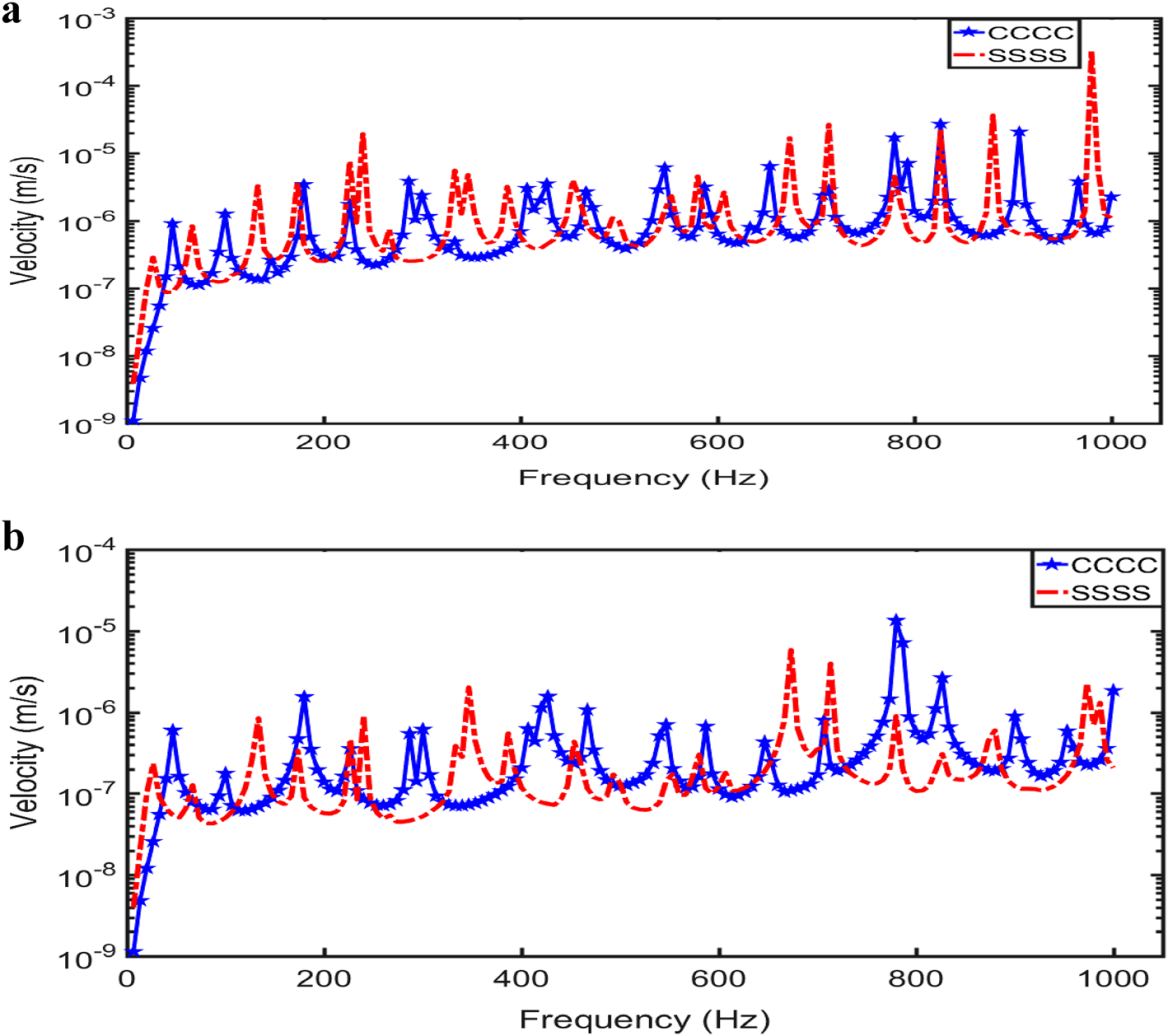
Velocity-frequency response comparison of two finite element models with the same modelling parameters but different boundary conditions for sound source generated at (**a**) NBC, (**b**) FBC.

Figure 7 compares the velocity-frequency responses of the two numerical results at two source locations. It is observed that at the same source location (i.e., NBC or FBC), with the different boundary conditions, the velocity amplitudes of the vibrating square panel are slightly different while the frequencies of the square panel with CCCC condition shift more to the right than the square panel with SSSS boundary condition.

**Figure 8:**
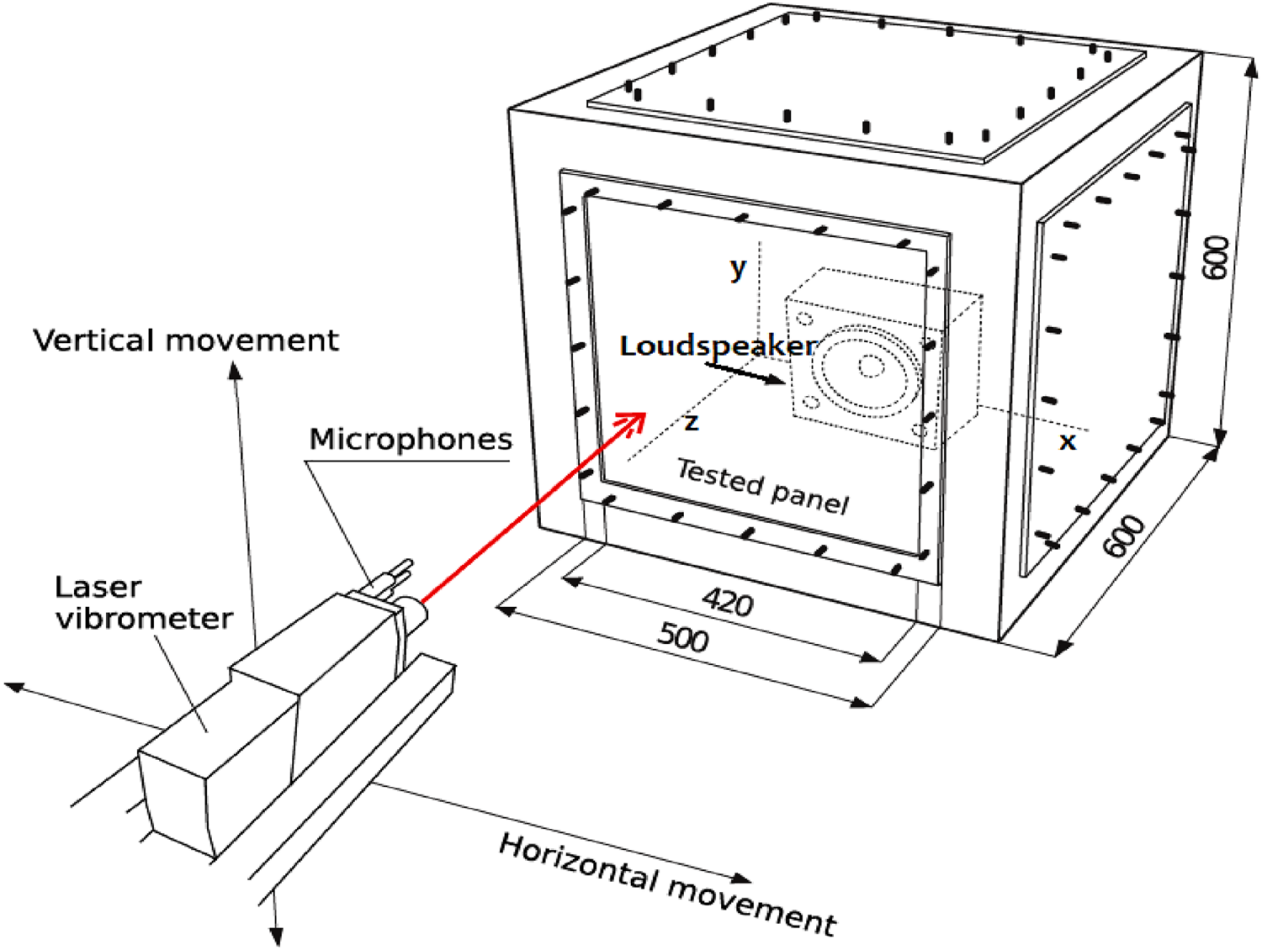
Illustrative diagram of the experimental set-up for acoustic radiation measurements.

**Figure 9:**
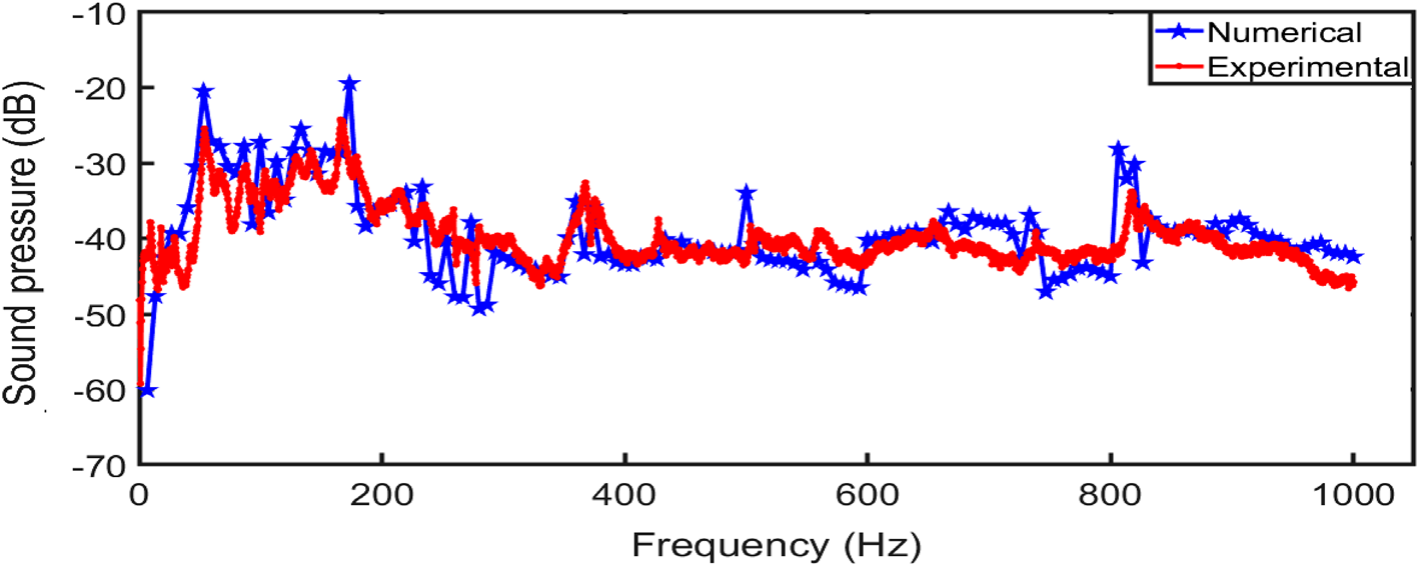
Comparison of the acoustic radiation in terms of averaged sound pressure between experimental measurement and numerical approximation.

For the acoustic radiation measurements, the microphones (Beyerdynamic MM1) were mounted on an automatic positioning system developed by the authors, capable of moving the microphones in horizontal and vertical axes as illustrated in Fig. 8. The sound pressure was measured with the microphones over a measurement grid that was 1.00 m wide and 0.64 m high and located at a distance of 0.1 m from the plate surface, with an interval between the measurement points of 0.04 m (this gives 26 × 17 measurement points, which is a total of 442 points). The mean values were obtained by averaging over the described measurement grid. Figure 9 compares the averaged sound pressure between the experimental measurements and finite element approximations. The acoustic radiation of the numerical solution shows good correlation with the measured results. Hence, it provides a basis to perform further comparative analyses of the influence of sound source location on *STL*, *SPL* and far-field directivity as discussed elaborately in the following sections.

## Model vibro-acoustic response and discussion

In this section, various finite element solutions of the vibro-acoustic problems are discussed. The modelled lightweight square panel with CCCC boundary condition is used for the numerical examples and analyses. Six location categories of the sound source (i.e., loudspeaker) are used for the analyses of the study. In location category one (LC1), the sound source is positioned at the centre and placed on the base of the rigid casing with near (NBC), middle (MBC) and far (FBC) distance locations. In location category two (LC2), the sound source is suspended at the mid-centre of the rigid casing with near (NCC), middle (MCC) and far (FCC) distance locations. In location category three (LC3), the sound source is placed on the base and at the edge of the rigid casing with near (NBE), middle (MBE) and far (FBE) distance locations from the square panel. Furthermore, in location category four (LC4), the sound source is fixed from a near location to the square panel but positioned at the base (NBC), mid-centre (NCC) and edge (NBE) of the rigid casing. In location category five (LC5), the sound source is fixed at a middle location from the square panel but positioned at the base (MBC), mid-centre (MCC) and edge (MBE) of the rigid casing. Lastly, in location category six (LC6), the sound source is fixed at a far location from the square panel but positioned at the base (FBC), mid-centre (FCC) and edge (FBE) of the rigid casing. The influence of these different locations of a loudspeaker on the vibro-acoustic emission indexes are herein analyzed.

### Effect of sound source locations on $\mathrm{STL}$

**Figure 10:**
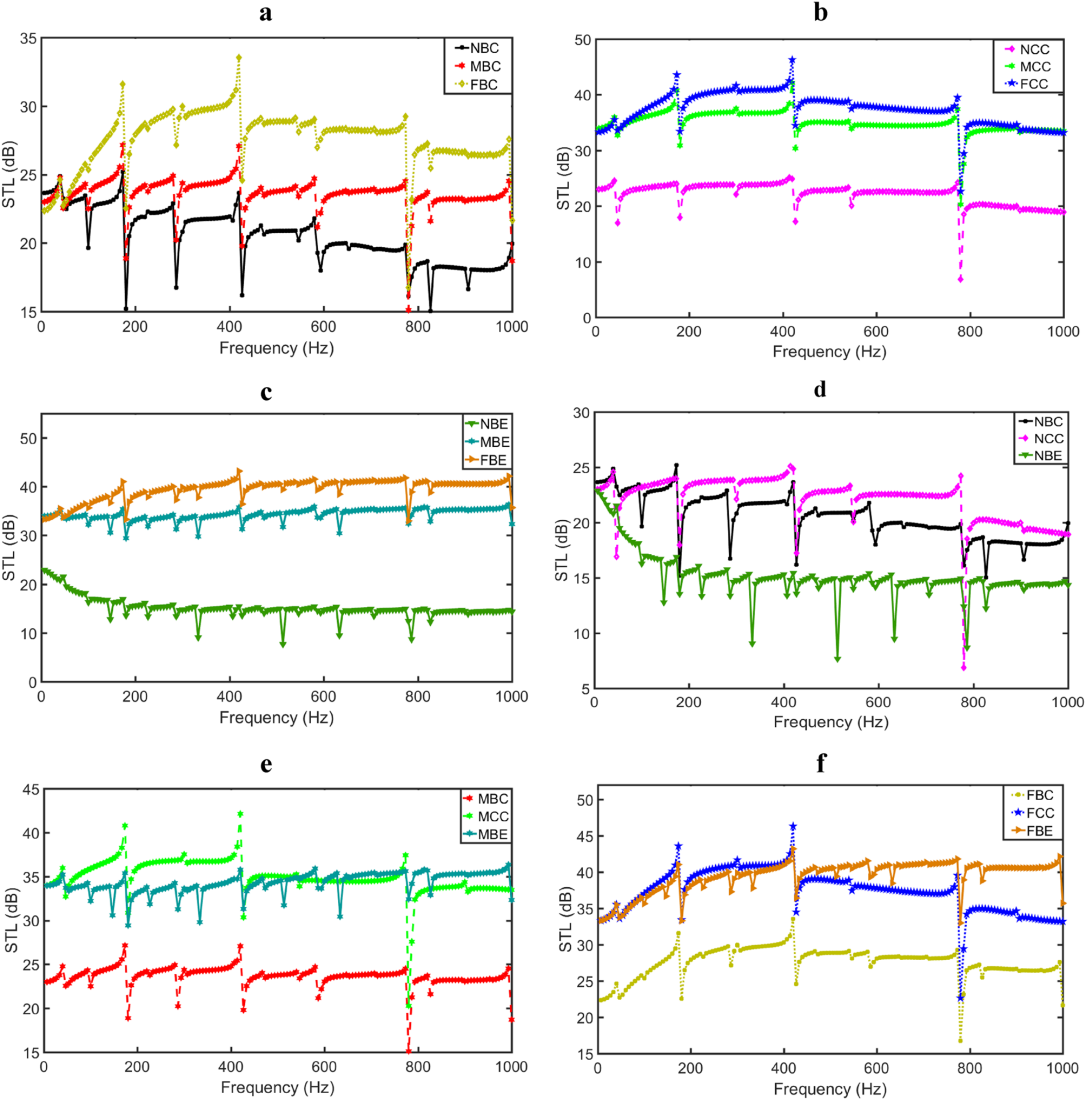
Transmission loss comparison of six location categories of the sound source (**a**) LC1, (**b**) LC2, (**c**) LC3, (**d**) LC4, (**e**) LC5, (**f**) LC6.

Figure 8 represents the results of the transmission loss of the aluminium panel for different locations of the loudspeaker. The sound transmission loss ($STL$) results show that $STL$ is greatly influenced by the locations where the sound waves emanate. For the LC1, as seen in Fig. 10a, when the sound wave is generated at FBC, the $STL$ of the panel is seen to increase. This result is expected. The farther the distance of the primary sound waves to the vibrating panel, the increase in the $STL$ of the panel. Moreover, it is observed that at a very low frequency (i.e., 0–35 Hz), the $STL$ values increased as the sound source gets nearer to the vibrating panel. Also, for this LC1 situation, it is seen that each of the first natural frequencies occur at approximately the same value. However, along the remaining frequency range, $STL$ increased with farther distance from the vibrating panel. Furthermore, the result shows that while the $STL$ due to sound waves generated at NBC decreases with increasing frequencies, the $STL$ due to sound waves generated at MBC is relatively constant along increasing frequency. The $STL$ results obtained due to sound waves generated at FBC increased at mid-frequencies and then begin to decrease slightly from mid to higher frequencies. For the LC2 case as depicted by Fig. 10b, it is seen that when the sound wave is generated at NCC, the $STL$ of the vibrating panel becomes very low compared to when the sound waves are generated at MCC and FCC. When the source of noise is at this point (i.e., NCE), for active noise control, more microphones will be needed to improve the noise reduction efficiency of the vibrating plate. Also, it is observed that with the LC2, the natural frequencies which can be seen from the peak and dip of the $STL$ curves occur at approximately the same points along the frequency range. In Fig. 10c, when the generated sound waves is located at the edge and near the vibrating panel (i.e., at NBE), the $STL$ of the panel is significantly reduced compared to when the sound waves are generated at MBE and FBE. It is also observed that at this LC3 positions, more peaks and dips are formed along the entire frequency range. This is the effect caused for the generated sound waves located near edge of the wall casing. In Fig. 10d, when the source is fixed at a near point location to the vibrating panel, it is seen that the $STL$ due to sound waves generated at NCC is mostly improved and consequently, the least number of actuators or sensors will be required to produce better transmission loss than those sound waves generated at NBC and NBE. However, for sound waves generated at NBE, more actuators will be required by the vibrating panel to increase the $STL$. In Fig. 10e, when the loudspeaker is located at the mid-centre of the casing, it is evident that the sound waves at MBC produce a very low $STL$ of the vibrating panel than the sound waves emanating at MBC and MCC locations. Lastly, Fig. 10f shows that at a far location of the sound waves, the sound wave emanating at FBE produced higher $STL$ along the mid to high frequency range compared to those of the sound waves generated at FCC and FBC. In this situation, more microphones will be expected to be used for active noise control. Similarly, more actuators/sensors will be expected to be used for active vibration control when the sound source originates at FBC than at FCE or FBE. In general, it is observed that by positioning the loudspeaker at the edge of the wall casing, the $STL$ of the vibrating panel do not necessarily reduce in value. In fact, sound waves generated at FBE and MBE show higher $STL$ at low and mid-frequency regions. Moreover, as the generated sound waves move away from the casing wall boundaries (i.e., at NBC, MBC, FBC), the frequency dips are observed to increase in amplitudes.

### Effect of sound source locations on sound pressure level

**Figure 11:**
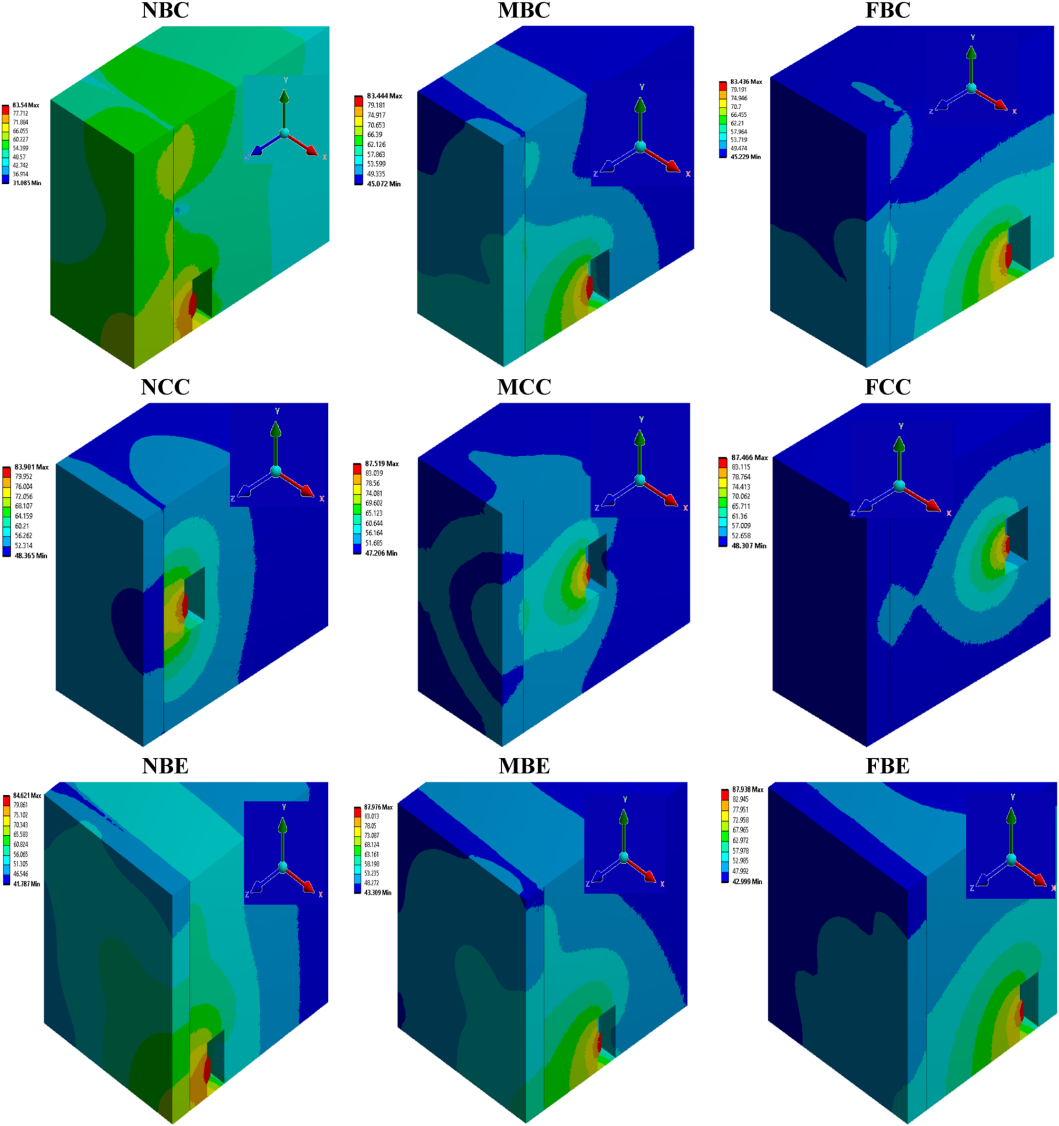
Sound pressure level distribution profile at frequency of 180 Hz for all sound source locations. The rationale for choosing this frequency is to fully capture the sound distribution at the radiated end of the panel where a microphone can be positioned for noise attenuation or active noise control which are typically carried out at low frequency.

Figure 11 represents the sound pressure level $(SPL)$ wave distribution at a low frequency of 180 Hz for the different locations of the loudspeaker with observation in the $xy$-plane along $z$-direction of the radiating panel. The figure shows that the radiated sounds for all locations of the sound source are completely different. Zones of quiet can be observed at the radiated end of the panel. The zones of quiet is the quiet zones formed at the radiated near field region of the vibrating aluminium panel for the different locations of the primary sound source (i.e., the loudspeaker). While the most zones of quiet are observed for sound waves generated at FCC, the least zones of quiet is experienced for sounds generated at NBC. For the LC1 case (i.e., at NBC, MBC, and FBC), it is observed that the most zones of quiet at the radiated surface of the square panel is sound generated at FBC, followed by that at MBC and lastly by that at NBC. For LC2, the most zones of quiet at the radiated surface is the sound source effect for sound location at FCE. For LC3, the zones of quiet at their radiated end is relatively compared. However, sound waves location at FBE gives the most zones of quiet compared to those at sound waves generated at MBE and NBE. For the LC4 case (i.e., NBC, NCC and NBE), the most zones of quiet at the radiating surface is observed by sound waves generated at NCC. At this radiated end of the panel, it is observed that a more quiet zone concentrates at the centre of the radiating panel. For LC5, it is observed that more zones of quiet is experienced when the sound wave is generated at MCC than those sound waves emanating from MBC and MBE. Lastly, for LC6, (i.e., at FBC, FCC and FBE), it is clearly seen that sound waves location at FCC produces the most quiet radiated noise zone followed by sound location at FBC and lastly by sound location at FBE. For each location of the generated sound waves, the $SPL$ wave distribution at the radiated surface can help to inform researchers on how to effectively position both actuators and microphones on and around the vibrating panel, respectively. For instance, in the LC2 case, actuators may be positioned on the radiating end of the vibrating panel where the recorded $SPL$ produced by sound waves generated at NCC, MCC, and FCC are 52.3 dB, 51.7 dB, and 52.7 dB, respectively. This will help to further reduce the noise for active vibration control^30^. Moreover, by using this information for active noise control, microphones can also be carefully positioned around the vibrating panel with respect to the primary sound source distribution and spectrum.

**Figure 12:**
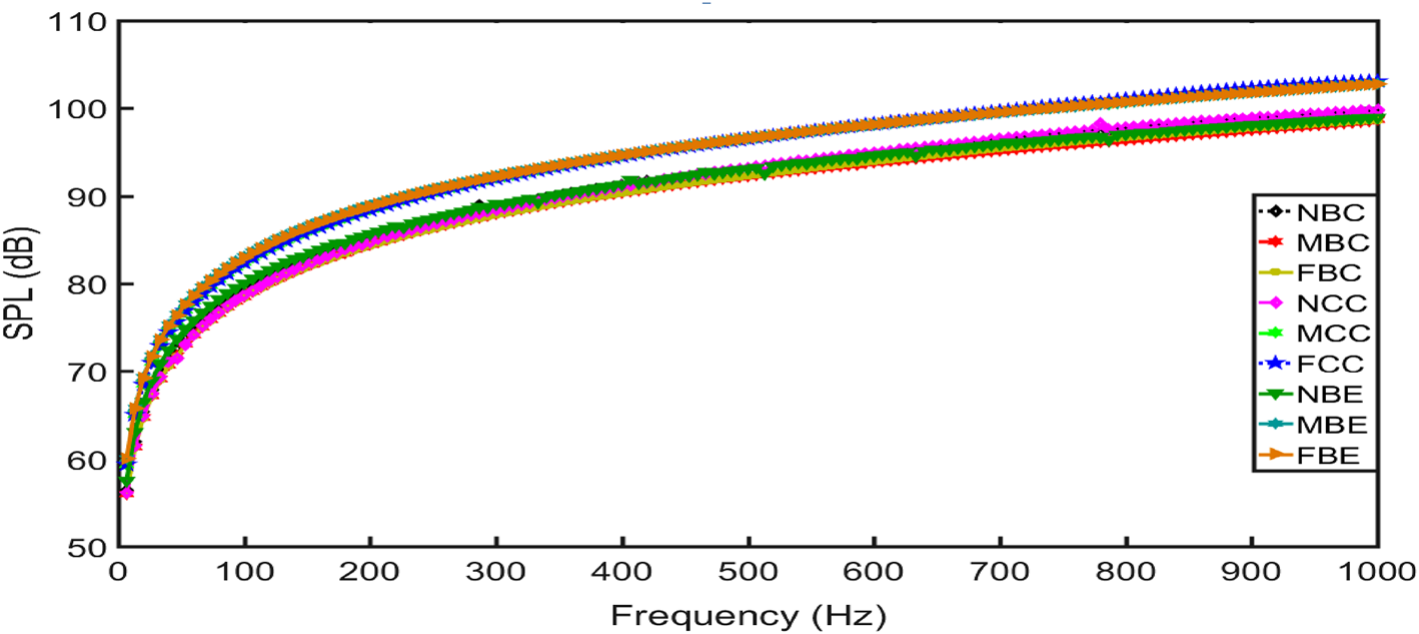
Maximum sound pressure level curve along the entire frequency range for all sound source locations.

Figure 12 shows the maximum $SPL$ along the entire frequency range for all sound source locations used in this study. At a higher frequency of 1000 Hz, the maximum amplitude of $SPL$ for sound waves generated at NBC, MBC and FBC are 99.26 dB, 98.70 dB and 98.61 dB, respectively. It is seen that at this frequency, the latter $SPL$ result indicates that sound waves generated at FBC produces the most noise reduction level of the vibrating panel than those generated at MBC and NBC.

### Effect of sound source locations on far-field directivity

**Figure 13:**
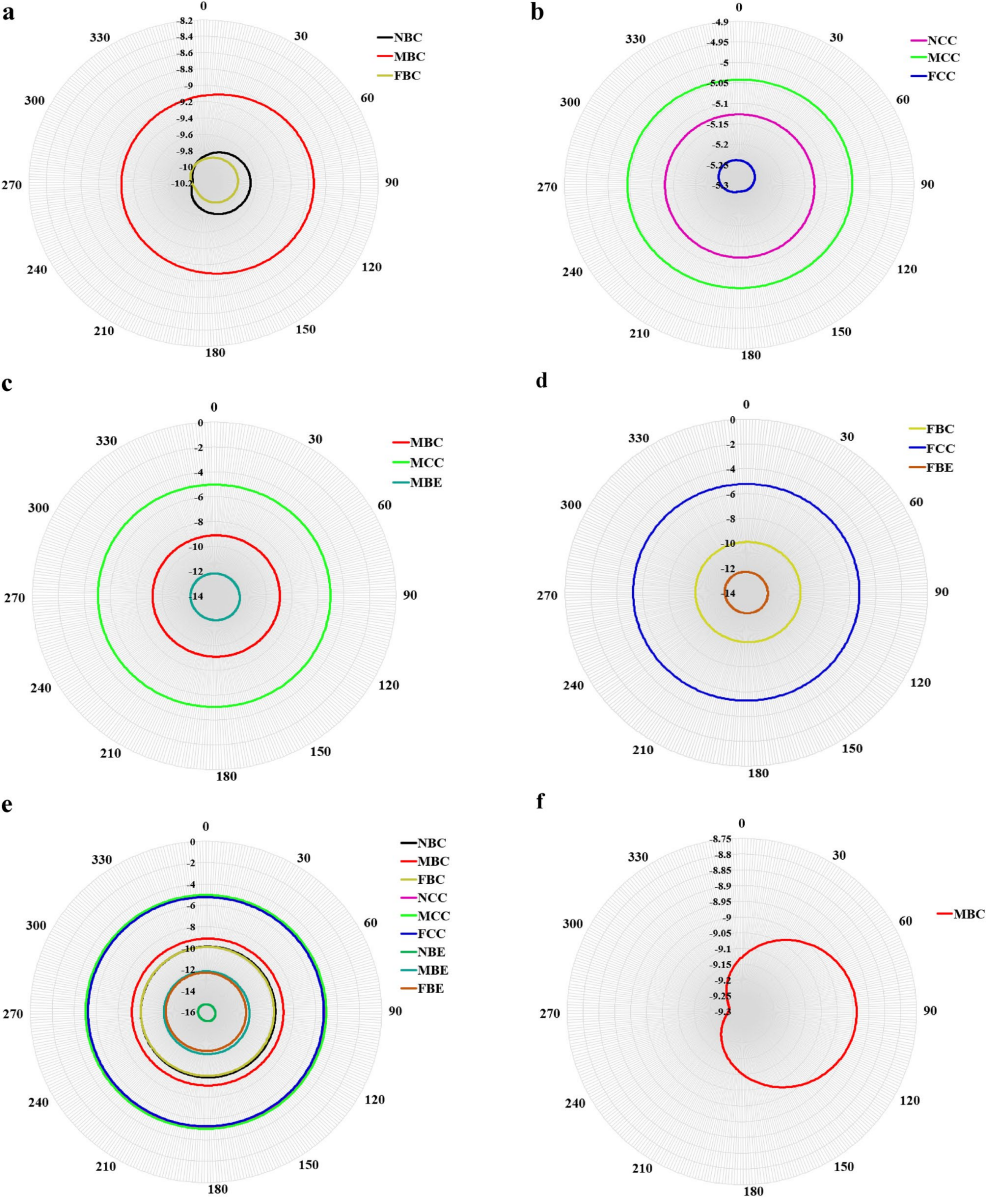
Far-field directivity of sound pressure comparison for different location categories of the sound source at $f=146$ Hz (**a**) LC1, (**b**) LC2, (**c**) LC5, (**d**) LC6, (**e**) all locations, (**f**) MBC.

**Figure 14:**
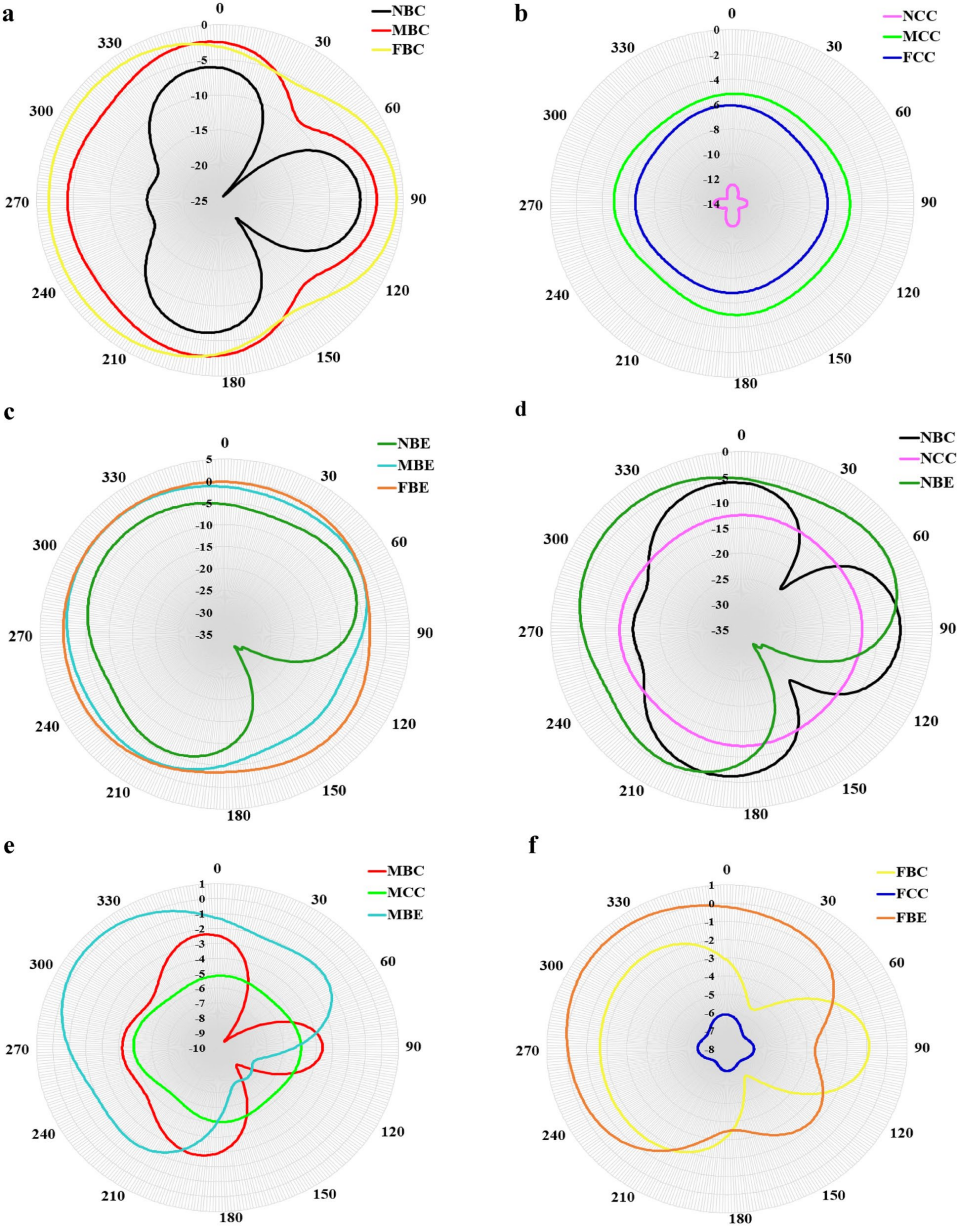
Far-field directivity of sound pressure comparison for different location categories of the sound source at $f=1000$ Hz (**a**) LC1, (**b**) LC2, (**c**) LC3, (**d**) LC4, (**e**) LC5, (**f**) LC6.

The far-field directivity plots of the radiated sound pressure at different location categories of the loudspeaker, evaluated at two frequencies $f$ and at a far-field distance ${R}_{0}=10000$ mm, are presented in Figs. 13 and 14. For all results, the observation point is $0\le \beta \le {360}^{0}$ at polar angle $\theta =\pi /6$. For Fig. 13, the plots are evaluated at the fourth vibration mode of frequency $f=146$ Hz. At this frequency, it is seen that the amplitude of the far-field directivity of the radiated sound pressure of the square aluminium panel varies with different locations of the sound source. Also, it is generally observed that at low frequency regions, simple lobes (i.e., like monopoles, see Fig. 13f) are formed and the far-field directivity patterns of the radiated sound pressure vary smoothly along the circumferential angle with almost similar shapes for all the location categories of sound source as presented in Fig. 13a–d. However, due to the possibility of multimodal contribution during vibration of the square panel, the far-field directivity patterns become more complex at higher frequencies (i.e., at $f=1000$ Hz.) with different locations of the sound source as depicted in Fig. 14. At these frequencies, more irregular lobes are seen to be formed. Furthermore, as observed from these diagrams, none of the directivity patterns due to the different sound source locations, has the same shape.

### Effect of boundary conditions on far-field directivity

**Figure 15:**
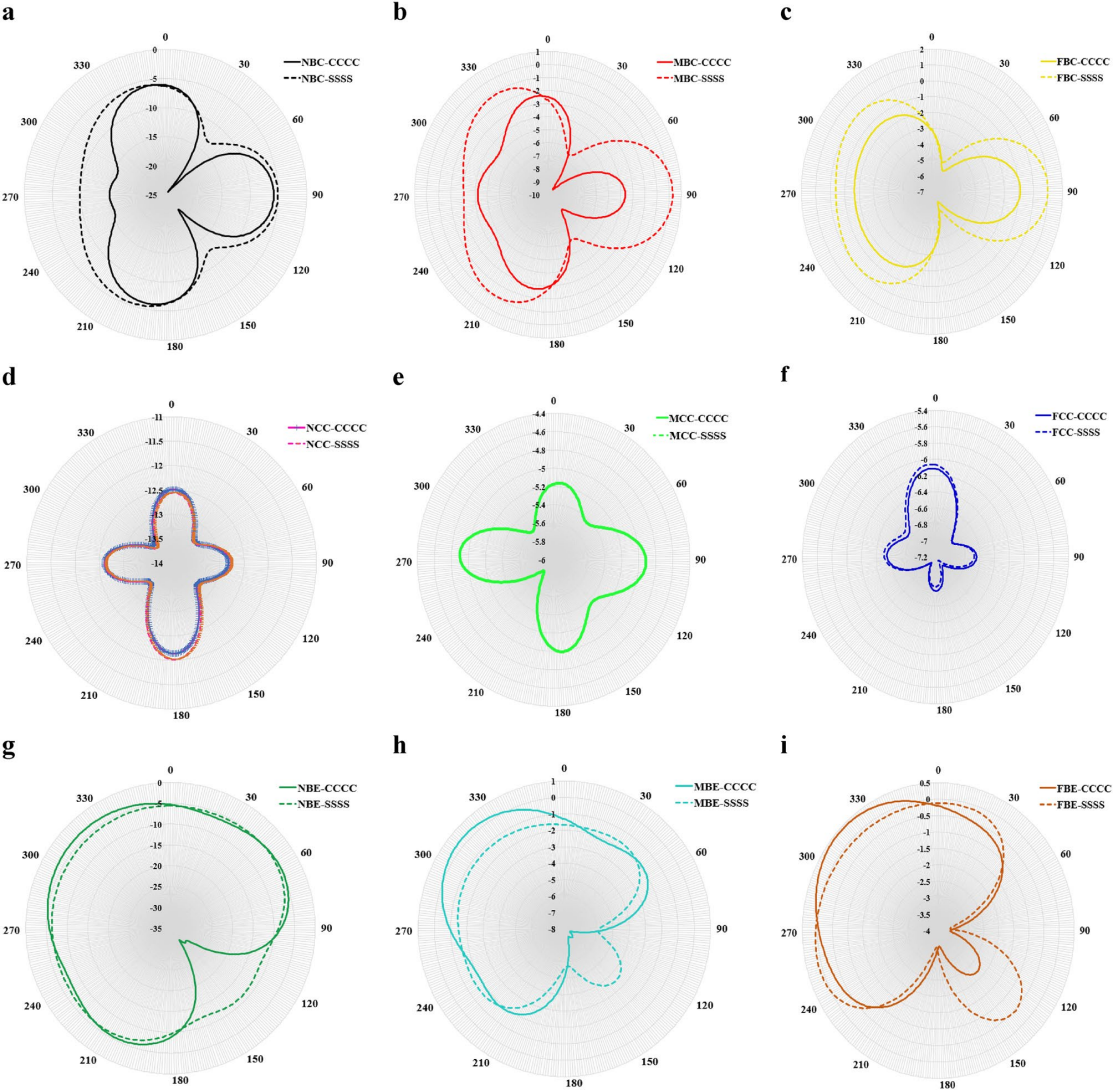
Boundary conditions comparison of the far-field directivity of sound pressure for different location of sound source at $f=1000$ Hz (**a**) NBC, (**b**) MBC, (**c**) FBC, (**d**) NCC, (**e**) MCC, (**f**) FCC, (**g**) NBE, (**h**) MBE, (**i**) FBE.

Figure 15 shows the influence of different boundary conditions BCs (i.e., CCCC and SSSS) of the vibrating panel on the far-field directivity of sound pressure for all locations of the sound source. The first, second and third rows show the far-field directivity when the sound source is placed at the bottom centre, mid-centre and bottom edge, respectively. The different directivity patterns of the sound pressure (i.e., those observed along the first and third rows of Fig. 15) show how the BCs of the vibrating panel influence its response at the acoustic far-filed condition. However, at mid-centre (i.e., those observed along the second row of Fig. 15) for each location of sound source, the different BCs given to the vibrating panel show no significant difference of the far-field directivity patterns. Moreover, along the mid-centre with source location at MCC (Fig. 15e), it is observed that the directivity patterns for the different BCs are approximately the same.

## Conclusion

Numerical modelling of the structural–acoustic response of lightweight square aluminium panel has been presented in this paper. Finite element method was used to model both the finite structural–acoustic domain and the far-field acoustic domain. The study considered the influence of changing locations of a primary sound source on the vibro-acoustic response of a square panel. Experimental results of the modal analyses of the aluminium square panel were used to validate the numerical approximation results. Major vibro-acoustic characteristic parameters such as the sound transmission loss, sound pressure level and far-field directivity of the sound pressure were obtained with respect to the different locations of the sound source. It was shown that the varied positions and locations of the primary sound wave emanating from a loudspeaker significantly influenced the efficacy of the vibrating panel to reduce noise. Sound waves which are generated at locations near the vibrating panel, lowers the efficacy of the panel to reduce noise. Also, as the generated sound waves move away from the vibrating panel, the potential of the structural panel to reduce noise increased. However, it was observed that when the generated sound waves was positioned close to the edge of the wall casing, the $STL$ of the vibrating panel is not necessarily reduced except when the generated sound waves was very close to the vibrating panel. Moreover, the variation of the zones of quiet at the radiated region as well as the radiated sound pressure, sound transmission loss and far-field directivity, have provided useful information on the best position of sensors or actuators for active vibration control. Also, the radiated sound obtained due to the different locations of the sound source can inform researchers of active noise control on the adequate number of secondary sources and their best positions with respect to their primary sound wave spectrum.
